# Concurrent congenital peritoneopericardial diaphragmatic hernia and bochdalek hernia in a neonate

**DOI:** 10.1186/2193-1801-3-290

**Published:** 2014-06-09

**Authors:** Jonathan F Bean, Chad A Kort, Jayant Radhakrishnan

**Affiliations:** Resident in Surgery, Division of Pediatric Surgery, Department of Surgery, University of Illinois, Chicago, IL USA; Ex-resident in Surgery, Division of Pediatric Surgery, Department of Surgery, University of Illinois, Chicago, IL USA; Professor Emeritus of Surgery & Urology, Division of Pediatric Surgery, Department of Surgery, University of Illinois, 1502 71st Street, Darien, IL 60561 USA

**Keywords:** Peritoneopericardial diaphragmatic hernia, Bochdalek hernia, Septum transversum

## Abstract

We present the first report of a neonate with, concurrent left sided Bochdalek hernia and peritoneopericardial diaphragmatic hernia.

## Case report

**Figure 1:**
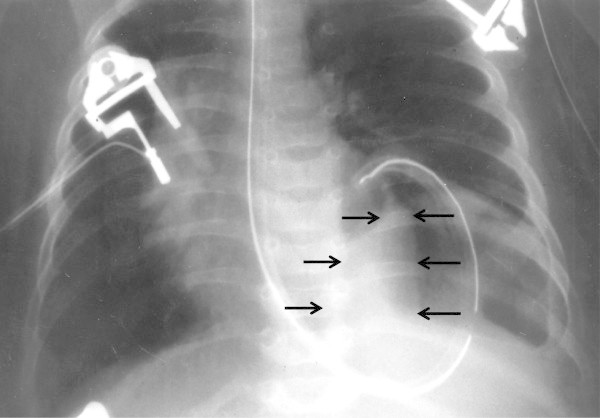
**Frontal chest radiograph demonstrating nasogastric tube in the left hemithorax with displacement of the mediastinum to the right.** Note the normal cardiac shadow. The left lobe of the liver is in the chest and is demarcated by black arrows.

**Figure 2:**
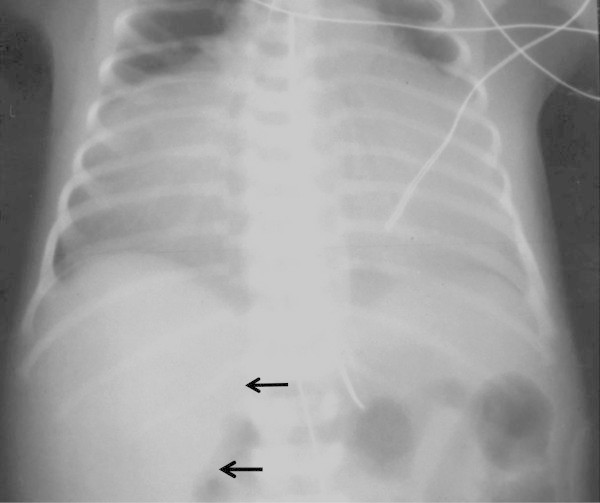
**Chest radiograph 24 hours after repair of Bochdalek hernia.** The grossly widened mediastinum, consisting of the mass and the heart, occupies almost the entire thorax. Only the right lobe of the liver is seen in the abdomen (marked by black arrows). The nasogastric tube and umbilical artery catheter are in place.

**Figure 3:**
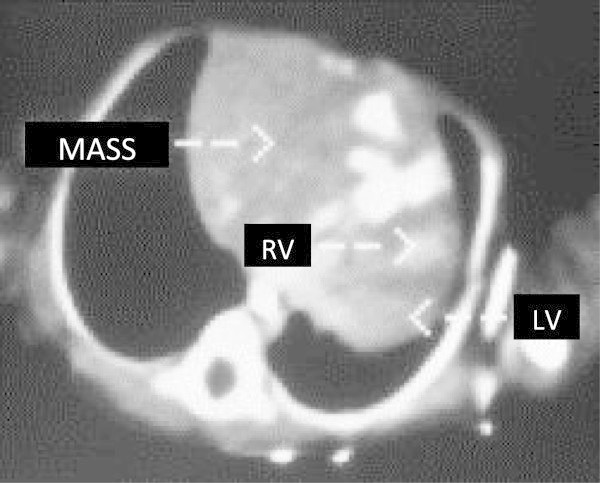
**CT scan of the chest demonstrates the mass (identified) to the right of the heart.** RV = Right Ventricle, LV = Left Ventricle.

**Figure 4:**
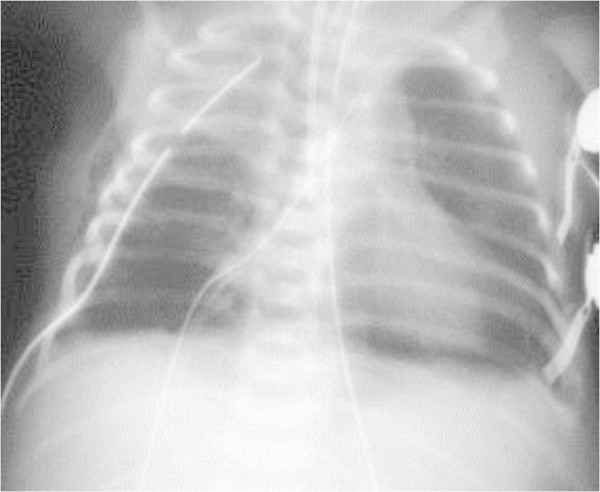
**Postoperative frontal radiograph after repair of the peritoneopericardial hernia.** Resolving atelectasis of the upper lobe of the right lung is apparent. The mediastinum and both leaves of the diaphragm are normal. The endotracheal tube, nasogastric tube, right chest tube and pericardial tubes are visible.

A 2,600 gram male was born at 37 weeks gestation with a diagnosis of left congenital diaphragmatic hernia made by prenatal ultrasonography. No other congenital abnormalities were found. The APGAR scores were 6 and 9 at 1 and 5 minutes, respectively. A frontal chest radiograph revealed the stomach in the left chest with shift of the mediastinum to the right. The heart was normal in shape and size. The left lobe of the liver was in the left chest (Figure 1). The child was stable at birth and continued to do well without cardiorespiratory support or evidence of right to left shunting hence he was operated upon on the first day of life. The hernia was approached *via* a left subcostal incision. Upon reduction of viscera a standard left Bochdalek diaphragmatic defect 2cm in diameter was noted. Primary repair of the defect was carried out easily without a patch and the abdomen was closed without creating a ventral hernia. Twenty four hours after surgery the infant began to require increasing respiratory and cardiac support. A chest radiograph revealed a massively widened mediastinum. The so called liver cut-off sign, in which the liver shadow is not visible to the left of the spine, was present but we missed it (Figure 2). The echocardiogram showed an effusion and a mass in the pericardium to the right of the heart. It was felt that the mass was probably an iatrogenic hematoma possibly due to the umbilical artery catheter. A computerized tomographic (CT) scan was carried out to better delineate it. The CT scan demonstrated the mass to have the same density as the liver (Figure 3). Since the diagnosis was in question the patient was re-operated through a right anterolateral thoracotomy to permit vascular or cardiac repair, if required. The pericardium was opened vertically, anterior to the right phrenic nerve. The defect in the central tendon had a well-defined tendinous margin circumferentially, the caudal pericardium was absent and there was no hernia sac. The herniated bare area of the liver was easily reduced from the thorax, however, the defect was large and it extended across to the left edge of the pericardium. Hence it was safer to repair the defect with a Gore-Tex patch (W.L.Gore & Associates, Flagstaff, AZ) attached to the abdominal surface of the diaphragm to avoid compressing the heart during the repair. This was achieved through a right subcostal incision (Figure 4). The patient had an uneventful post-operative course following the second operation.

## Discussion

In neonates, a left sided Bochdalek hernia is the most common congenital diaphragmatic hernia. It occurs in the posterolateral part of the diaphragm, rarely has a sac and is currently believed to develop as a result of defects in the posthepatic mesenchymal plate (PHMP) (Mayer et al. 2011). The presentation, diagnosis and management of Bochdalek hernias is well known and will not be repeated.

According to Wilson the rare congenital peritoneopericardial diaphragmatic hernia (PPDH) was first described by De Cardinal and Bourderou in 1903 (Wilson et al. 1947). This hernia traverses a defect in the central tendon, and it may be contained in a sac (Davies et al. 1993). The retrosternal or Morgagni hernia may also be intrapericardial and have a similar presentation; however, it passes through spaces adjacent to the xiphoid process at the junction of the anterior diaphragm with the chest wall (Akalin et al. 2004; Antinolo et al. 2010). The embryology of the central tendon defect is unclear. It is unlikely that the PPDH defect in the central tendon results from a complete absence of the septum transversum since in that case the liver and the ducts of Cuvier would not form (Skandalakis et al. 1994). However, it could result from a defective septum transversum (Yuan et al. 2003; Asahina et al. 2011) as lesser abnormalities of the liver such as absence of the suspensory ligament and elongated ductus venosus have been described in these patients (Casey & Hidden 1944; Wesselhoeft & DeLuca 1984). The presence of intrapericardial fat pads has also been blamed for development of the central tendon defect (Pomputius & Fisher 1966). It appears most likely that the lesion results from rupture of a septum transversum weakened by the rapid growth of the liver into it (Skandalakis et al. 1994).

Previously most patients with PPDH presented with respiratory distress and respiratory failure due to bilateral pulmonary hypoplasia. Further evaluation would reveal a widened mediastinum and pericardial effusion. The effusion is a transudate probably due to venous congestion in the incarcerated liver and it is believed these patients do not develop cardiac tamponade as the fluid accumulates gradually (Einzig et al. 1981). On frontal abdominal radiographs the liver shadow is not visible to the left of the spine, the so called “liver cut-off” sign. The ultrasound examination and CT scan demonstrate a solid mass in the pericardium contiguous with and having the same density as the liver. The mass may have the appearance of a three tiered snowman (Wesselhoeft & DeLuca 1984). With improvements in prenatal diagnosis, many patients are now diagnosed using fetal ultrasonography (Kanamori et al. 2005; Hara et al. 2007).

The defect is best repaired with a prosthetic patch which is most securely and satisfactorily attached to the abdominal surface of the diaphragmatic defect. The abdominal approach is therefore best for acute cases and when the diagnosis is clear. Diagnosis and repair can be carried out laparoscopically (Paci et al. 2005). In chronic cases, when adhesions may be present between the liver and the pericardium or the heart, an initial thoracic exposure seems to be safest (Kessler et al. 1991). The thoracic approach is also best if the diagnosis is in question since other intrapericardiac masses such as teratomas and cysts are best removed through the chest (Gross 1946).

This unusual patient creates a few problems for clinicians. First, this is the only report of two different types of congenital diaphragmatic hernia existing concurrently in the same patient. It is important to be aware that this rare association is possible. Secondly the liver did not herniate into the pericardium until intraabdominal pressure was elevated after repair of the Bochdalek hernia. Finally, the bare area of the liver protrudes in the peritoneopericardial hernia and it is encircled by the coronary ligament. Thus the defect in the diaphragm is not apparent by inspection or palpation of either leaf of the diaphragm.

## Abbreviations

CT scan: Computerized tomographic scan
PPDH: peritoneopericardial diaphragmatic hernia.
